# Time for Dynamic Assessment of Fitness in Acute Myeloid Leukemia

**DOI:** 10.3390/cancers15010136

**Published:** 2022-12-26

**Authors:** Raffaele Palmieri, Luca Maurillo, Maria Ilaria Del Principe, Giovangiacinto Paterno, Roland Bruno Walter, Adriano Venditti, Francesco Buccisano

**Affiliations:** 1 Department of Biomedicine and Prevention, Tor Vergata University, 00133 Rome, Italy; 2 Clinical Research Division, Fred Hutchinson Cancer Center, Seattle, WA 98109, USA; 3 Department of Medicine, Division of Hematology, University of Washington, Seattle, WA 98195, USA; 4 Department of Laboratory Medicine & Pathology, University of Washington, Seattle, WA 98195, USA; 5 Department of Epidemiology, University of Washington, Seattle, WA 98195, USA

Informed treatment decision-making in acute myeloid leukemia (AML) requires a comprehensive evaluation of all clinical and biological features that may affect the outcome with any given type or intensity of therapy. This process needs to encompass an assessment of both the clinical fitness of the patient and the biological characteristics of the disease. Clinical fitness refers to individual characteristics (e.g., age, performance status, and comorbidities) that may impact tolerance to a given therapy, whereas biological characteristics involve disease-related features (e.g., genetic/cytogenetic abnormalities, history of antecedent hematologic disorder or exposure to chemotherapeutics or radiation) that may affect the sensitivity/resistance to a particular treatment modality [1]. However, despite the increasing number of scoring systems to help assess medical fitness and eligibility/ineligibility for active therapy (Table 1), there is still no consensus on how to best define fitness, nor which parameters should be taken into account for its determination [2]. Standardizing this evaluation process appears critical since fitness assessment has progressively taken a central role in registrational clinical trials, especially in the setting of patients considered unsuitable for “standard” intensive approaches. Whichever the preferred tool, the goal is to identify therapies that may (1) worsen age-dependent frailties, (2) cause organ damage due to pre-existing comorbidities, (3) be difficult to comply with, due to clinical or social conditions, or (4) be unlikely to be successful because of adverse cytogenetic/molecular disease features [3].

Moreover, such a careful evaluation should be performed at multiple time points during the therapeutic course, to intercept possible changes in features that are patient- and/or disease-related. The practical application of dynamic assessment of fitness may be difficult, especially in terms of the identification of the most appropriate time points at which re-evaluation should be conducted. In this view, we suggest that, during the treatment course, fitness re-evaluation should be performed whenever patient- and/or disease-related parameters may have changed in a way that treatment modality recalibration is required.

A comprehensive assessment of fitness and biological characteristics is performed at the time of AML diagnosis to inform the initial treatment selection. At diagnosis, AML is frequently associated with a deterioration of patients’ general conditions, characterized by signs and symptoms such as fatigue, dyspnea, and a decline in overall performance status. The magnitude of these signs and symptoms depends on features of the AML (e.g., disease burden, extramedullary disease manifestations, growth dynamic) and can be exacerbated by pre-existing medical conditions. A reliable evaluation of clinical fitness requires that vigorous supportive therapy is established at the time of diagnosis to relieve disease-related complications. This may avoid overtreatment (when signs and symptoms are mistakenly attributed to the disease, underestimating pre-existing conditions) or undertreatment (when concurrent AML conditions, with an influence on general status, are not properly identified) [1]. The decision-making process might become particularly challenging if a discordance exists between the patient’s and disease features. It may occur that a patient is considered eligible for intensive chemotherapy, and yet the underlying AML biology indicates low probabilities of response.

During the treatment course, dynamically re-evaluating clinical fitness is essential in circumstances where the condition of the patient has changed. Such changes are more often pejorative, caused by treatment-related complications (e.g., infections, organ failures, etc.), but improvement of the patient’s functional status is also possible (e.g., if health was significantly impacted by disease-related problems). For the subset of patients with changing health status during therapy, a clinical fitness reassessment is intended to adjust the treatment type/intensity as appropriate, to optimally align the therapy with current patient fitness and disease characteristics. As an example, an oral formulation of azacitidine has shown to improve survival when administered as maintenance therapy in patients in complete remission after intensive induction chemotherapy, and that would typically be considered for allogeneic hematopoietic cell transplantation from a disease biology perspective, but eventually could not for medical or logistic reasons [7]. Conversely, patients considered unfit for intensive treatments at initial assessment may, once treatment with a lower-intensity therapy has begun, improve their overall level of fitness and become better candidates for more intensive treatment approaches. Doing so may secure greater curative potential, minimizing excessive toxicity. In a posthoc analysis of two multicenter trials, adults with newly diagnosed AML considered ineligible for intensive chemotherapy were randomized to azacitidine, decitabine, or low-dose cytarabine, plus venetoclax versus placebo [8,9]. Approximately 10% of the patients ultimately underwent allogeneic HCT after achieving complete remission [9]. This observation suggests the possibility that fitness status at diagnosis was substantially affected by disease-related symptoms rather than pre-existing disease-unrelated organ function limitations or performance status impairments. In this subset, re-assessment of the patient’s fitness and disease biology supported a change in treatment strategy, possibly improving outcomes in some.

In addition to the dynamic assessment of the medical fitness, the patient’s AML disease biology will also require serial assessments due to disease evolution, by itself or under the pressure of AML therapeutics, which may result in changes to clonal and sub-clonal disease architecture. It is not uncommon to detect leukemic clones that carry specific mutations at disease relapse or progression when they were not originally detected at AML presentation. Nowadays, with the availability of several new targeted agents for routine clinical use [10], the importance of characterizing a new, emerging leukemic population with a specific “biological fingerprint” is not limited to prognostication but extends to therapeutic implications. Accordingly, the assumption that genetic/cytogenetic profile may vary at disease relapse underlines the prognostic relevance of a dynamic complete molecular (re)testing over time to optimize salvage treatment strategy [11].

With the increasing availability of targeted agents, frontline treatments may be selected primarily based on disease characteristics rather than medical fitness. For instance, some emerging data may suggest that specific lower-intensity therapies (such as venetoclax in combination with an azanucleoside) may be as effective as intensive chemotherapy and yet better tolerated [12]. As a future perspective, a treatment tailored to the biologic profile of AML more than age or fitness could be adopted to “spare toxicities” during the remission induction, thus increasing the tolerability to subsequent intensive therapies (including HCT).

The need for a timely definition of clinical fitness and biological characteristics challenges the traditional doctrine that non-M3 AML is to be always considered a medical emergency. Indeed, several studies, including a large recent study from a German SAL-AML registry, indicate that the time from AML diagnosis to treatment does not affect the likelihood of response in a majority of patients [13,14,15]. Except for clinically unstable patients in need of urgent treatment initiation because of specific problems such as disseminated intravascular coagulopathy (DIC) or hyperleukocytosis/leukostasis, it may be feasible to wait, not only for genetic test results (as originally displayed in the study), but also to properly identify, and appropriately manage concurrent and transient medical conditions that may influence therapy delivery, tolerance, and compliance [15].

Although there’s still no consensus on how fitness should be measured, the fact that clinical and biological features may change under therapeutic pressure in AML is unquestioned. In line with this, the decision-making process of AML treatment should not ignore the dynamic clinical fitness and AML biology assessment at specific informative time points throughout the treatment course and specificallyat baseline before treatment allocation, after first-line therapy both in responding and not responding patients to optimize post-remission and salvage therapy, respectively, and at any time during follow up to select the best therapeutic approach in patients experiencing relapse (Figure 1).

## Figures and Tables

**Figure 1:**
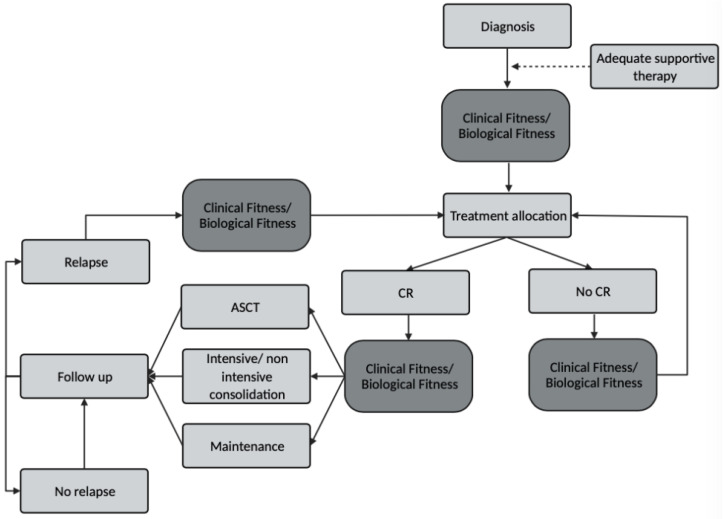
Critical time points for Clinical and Biological Fitness assessment.

**Table 1:** Prognostic algorithms for fitness assessment.

Fitness Score	Aim	Clinical Prognosticators	Biological Prognosticators	Reference
Treatment-related mortality (TRM)	To predict 28-days treatment-related mortality after induction chemotherapy	PS Age Albumin Creatinine	Secondary vs De novo Leukemia WBC count Platelet count %Blast peripheral	Walter et al.; J. Clin. Oncol. (2011) [4]
Acute myeloid leukemia composite model (AML-CM)	To predict the impact of a given treatment intensity based on clinical and biological parameters	Age Arrhythmia, Cardiac dysfunction Heart valve disease Inflammatory bowel disease, Diabetes, Peptic ulcer Cerebrovascular disease Psychiatric disturbance Obesity Infection Rheumatologic comorbidity Renal dysfunction Pulmonary comorbidity Prior malignancy Hepatic function Hypoalbuminemia < 3.5 g/dL LDH level	Thrombocytopenia ELN 2017 risk (favorable, intermediate, adverse)	Sorror et al.; Blood (2021) [5]
Geriatric assessment	To offer a comprehensive geriatric/quality of life assessment aside from established disease-specific variables	Cognition Psychological function (CES-D) Physical function (ADL, IADL, PAT-D, Mobility Sub-scale, PAT-D 6-mo recall, SPPB). Grip strength HCT-CI	None	Klepin et al.; Blood (2013) [6]
SIE/SIES/GITMO	To select treatment intensity based on a multi-organ functional evaluation, regardless of disease-related factors	Age PS Cardiac function Pulmonary function Renal function Infection, Psychiatric comorbidities, Other not classified	None	Ferrara et al.; Leukemia (2013) [3]

Abbreviations: PS, Performance Status; WBC, white blood cells; ELN, European Leukemia Net; CES-D, Center for Epidemiologic Studies Depression Scale; ADL, Activities of Daily Living; IADL, Instrumental Activities of Daily Living Scale; PAT-D, Pepper Assessment Tool for Disability; SPPB, Short Physical Performance Battery; and HCT-CI, Hematopoietic cell transplantation–specific comorbidity index.

## References

[1] Palmieri R., Paterno G., De Bellis E., Mercante L., Buzzatti E., Esposito F., Del Principe M.I., Maurillo L., Buccisano F., Venditti A. (2020). Therapeutic Choice in Older Patients with Acute Myeloid Leukemia: A Matter of Fitness. Cancers.

[2] Döhner H., Wei A.H., Appelbaum F.R., Craddock C., DiNardo C.D., Dombret H., Ebert B.L., Fenaux P., Levine R.L., Löwenberg B. (2022). Diagnosis and management of AML in adults: 2022 recommendations from an international expert panel on behalf of the ELN. Blood J. Am. Soc. Hematol..

[3] Ferrara F., Barosi G., Venditti A., Angelucci E., Gobbi M., Pane F., Tosi P., Zinzani P., Tura S. (2013). Consensus-based definition of unfitness to intensive and non-intensive chemotherapy in acute myeloid leukemia: A project of SIE, SIES and GITMO group on a new tool for therapy decision making. Leukemia.

[4] Walter R.B., Othus M., Borthakur G., Ravandi F., Cortes J., Pierce S.A., Appelbaum F.R., Kantarjian H.A., Estey E.H. (2011). Prediction of Early Death After Induction Therapy for Newly Diagnosed Acute Myeloid Leukemia With Pretreatment Risk Scores: A Novel Paradigm for Treatment Assignment. J. Clin. Oncol..

[5] Sorror M.L., Storer B.E., Fathi A.T., Brunner A.M., Gerds A.T., Sekeres M.A., Mukherjee S., Medeiros B.C., Wang E.S., Vachhani P. (2021). Multisite 11-year experience of less-intensive vs intensive therapies in acute myeloid leukemia. Blood.

[6] Klepin H.D., Geiger A.M., Tooze J.A., Kritchevsky S.B., Williamson J.D., Pardee T.S., Powell B.L., Ellis L.R. (2013). Geriatric assessment predicts survival for older adults receiving induction chemotherapy for acute myelogenous leukemia. Blood.

[7] Wei A.H., Döhner H., Pocock C., Montesinos P., Afanasyev B., Dombret H., Ravandi F., Sayar H., Jang J.-H., Porkka K. (2020). Oral Azacitidine Maintenance Therapy for Acute Myeloid Leukemia in First Remission. N. Engl. J. Med..

[8] Dinardo C.D., Jonas B.A., Pullarkat V., Thirman M.J., Garcia J.S., Wei A.H., Konopleva M., Döhner H., Letai A., Fenaux P. (2020). Azacitidine and Venetoclax in Previously Untreated Acute Myeloid Leukemia. N. Engl. J. Med..

[9] Pratz K.W., Dinardo M.C.D., Arellano M.L., Letai A.G., Thirman M., Pullarkat V.A., Roboz G.J., Becker P.S., Hong W.-J., Jiang Q. (2019). Outcomes after Stem Cell Transplant in Older Patients with Acute Myeloid Leukemia Treated with Venetoclax-Based Therapies. Blood.

[10] Döhner H., Wei A.H., Löwenberg B. (2021). Towards precision medicine for AML. Nat. Rev. Clin. Oncol..

[11] Vosberg S., Greif P.A. (2019). Clonal evolution of acute myeloid leukemia from diagnosis to relapse. Genes Chromosom. Cancer.

[12] Chen S., Xie J., Yang X., Shen H., Cen J., Yao L., Hu X., Wu Q., Zhang J., Qiu H. (2021). Venetoclax Plus Decitabine for Young Adults with Newly Diagnosed ELN Adverse-Risk Acute Myeloid Leukemia: Interim Analysis of a Prospective, Multicenter, Single-Arm, Phase 2 Trial. Blood.

[13] Sekeres M.A., Elson P., Kalaycio M.E., Advani A.S., Copelan E.A., Faderl S., Kantarjian H.M., Estey E. (2009). Time from diagnosis to treatment initiation predicts survival in younger, but not older, acute myeloid leukemia patients. Blood.

[14] Bertoli S., Bérard E., Huguet F., Huynh A., Tavitian S., Vergez F., Dobbelstein S., Dastugue N., Mas V.M.-D., Delabesse E. (2013). Time from diagnosis to intensive chemotherapy initiation does not adversely impact the outcome of patients with acute myeloid leukemia. Blood.

[15] Röllig C., Kramer M., Schliemann C., Mikesch J.H., Steffen B., Krämer A., Bornhäuser M., Thiede C., Wass M., Heits F. (2020). Does time from diagnosis to treatment affect the prognosis of patients with newly diagnosed acute myeloid leukemia?. Blood.

